# Prognostic competence as a criterion for the mental health of primary schoolchildren with psychological development disorders

**DOI:** 10.1192/j.eurpsy.2022.1782

**Published:** 2022-09-01

**Authors:** T. Artemyeva, V. Mendelevich, A. Akhmetzyanova

**Affiliations:** 1 Institute of Psychology and Education, Department Of Psychology And Pedagogy Of Special Education, Kazan, Russian Federation; 2 Kazan State Medical University, Department Of Medical And General Psychology, Kazan, Russian Federation; 3 Kazan Federal University, Department Of Psychology And Pedagogy Of Special Education, Kazan, Russian Federation

**Keywords:** predictive competence, development disorder, primary school student

## Abstract

**Introduction:**

Younger schoolchildren with psychological development disorders have low cognitive activity, insufficient development of basic school skills, and a low level of educational motivation. In accordance with the requirements of the educational program for students with psychological development disorders, it is important to develop the ability to predict the results of their actions.

**Objectives:**

The study of predictive competence in primary schoolchildren with psychological development disorders.

**Methods:**

The study involved 60 children aged 8-10 years with a psychological development disorder. To study predictive competence, the methodology “The ability to predict in situations of potential or real violation of social norms” was used.

**Results:**

The study revealed a low level of the cognitive and speech-communicative spheres of prognostic competence development in primary schoolchildren with psychological development disorders, as well as a deficit in prediction in the field of learning, which includes educational cooperation and educational communication of the child. Generalized statements, a passive position in future situations and pessimistic attitudes prevailed in the predictions of schoolchildren when constructing an image of the future. For schoolchildren with psychological development disorders, the prognosis is presented by monosyllabic answers, with the observable poverty of speech utterances.

**Conclusions:**

The features of prognostic competence revealed in the study make it possible to develop individual programs for the development of the prognostic abilities of schoolchildren with psychological development disorders. This paper has been supported by the Kazan Federal University Strategic Academic Leadership Program.

**Disclosure:**

No significant relationships.

